# Short versus Long Cephalomedullary Nailing in Osteoporotic Intertrochanteric Femur Fractures

**DOI:** 10.5704/MOJ.2603.013

**Published:** 2026-03

**Authors:** EM Zulkifli, SK Chandrasekaran

**Affiliations:** National Orthopaedic Centre of Excellence for Research and Learning (NOCERAL), Department of OrthopaedicSurgery, Universiti Malaya, Kuala Lumpur, Malaysia

**Keywords:** intertrochanteric femur fracture, osteoporotic fracture, long cephalomedullary nail, short cephalomedullary nail

## Abstract

**Introduction:**

This study compares the perioperative outcome of short versus long cephalomedullary nailing in the treatment of osteoporotic intertrochanteric femur fracture.

**Materials and methods:**

This is a retrospective cohort study in which 220 patients were reviewed, with 138 patients treated with long nails and 82 patients with short nails.

**Results:**

There were no significant differences between the two groups in terms of fracture union rate, mortality rate, or rates of surgical site infection, screw cut-out, implant failure, periprosthetic fracture, and revision surgery. However, patients treated with short cephalomedullary nails had significantly shorter operating times.

**Conclusion:**

This study concluded that both the long and short cephalomedullary nails are safe and reliable options in treating intertrochanteric femur fractures. However, the long nail does not provide extra protection against periprosthetic fractures as previously believed, and it has the disadvantage of longer operating time. Therefore, there is no additional clinical benefit in using the long cephalomedullary nail compared to the short cephalomedullary nail in treating osteoporotic intertrochanteric femur fractures.

## INTRODUCTION

Trochanteric fractures (AO classification 31A) are extracapsular hip fractures for which good fixation can be achieved by using either an extramedullary or an intramedullary device^1,2^. The use of an intramedullary device, however, has gained popularity^3^, likely due to its biomechanical superiority^4,5^, especially in unstable fractures^2^. However, some research indicates an intramedullary device, especially the short cephalomedullary nail (CMN), has a significant risk of a secondary fracture around the nail^6-8^. This is due to the formation of stress-riser at the tip of the nail^8,9^. The occurrence of this secondary fracture has been shown to be less in those treated with long CMN^1,6-8,10,11^. This finding has led surgeons to favour the use of long CMNs in the fixation of trochanteric fractures in osteoporotic bones as these implants are thought to protect the whole length of the bone^3,6,12^.

On the other hand, there are a few studies that have revealed no significant difference in the biomechanical^13^ and clinical outcomes between short and long CMNs. Both types of nails yield comparable rates of periprosthetic fractures, implant-related complications, and reoperations^14-16^. Furthermore, short CMNs may give the advantage of less blood loss, shorter operating time^10,14-18^ and lower costs of implant and hospital stay^17,18^.

At this centre, trochanteric fractures are usually treated with intramedullary devices. With the current background of conflicting data, the choice of the nail length in a trochanteric fracture without subtrochanteric extension is usually based on the quality of the bone and surgeon’s preference.

The study hypothesises that the short CMN is equivalent to the long CMN in terms of clinical outcomes when treating intertrochanteric femur fractures. The findings of this study may guide the choice of the length of the CMN used for trochanteric femur fractures in the future.

## MATERIALS AND METHODS

This research was a retrospective cohort study to compare the outcome between short and long cephalomedullary nailing in the treatment of intertrochanteric fractures of the femur. Ethical approval was obtained from the institutional review board. Patients were identified through the hospital database, and their electronic medical records were reviewed.

The inclusion criteria for this study were patients with osteoporotic intertrochanteric femur fractures (AO classification 31A) treated with either a short or a long CMN between January 2015 and December 2019 in our hospital, and patients with minimum length of follow-up of 12 months, with the exception for patients who had complications or died after the treatment, for whom no minimum length of follow-up was required. Exclusion criteria included patients with pathological fractures unrelated to osteoporosis, fractures with subtrochanteric extension, other ipsilateral femur fractures, or a history of ipsilateral femur or hip pathology or surgery

The patients were divided into two cohorts based on whether they were treated with a short or a long CMN. Data on patient, fracture, and implant characteristics were collected. Intra-operative and post-operative outcomes including operating time, blood loss, change in haemoglobin level, blood transfusion, intra-operative complications, allowance for weight bearing after surgery, length of hospital stay, fracture union, and post-operative complications were compared between the two groups.

Continuous variables were described using median with interquartile range (IQR) and categorical variables were presented as numbers and percentages. The data collected were also statistically analysed and compared. Kolmogorov-Smimov and Shapiro-Wilk tests of normality were done on all continuous variables. The Mann-Whitney test (for skewed distribution) or Independent T test (for normal distribution) was used to compare continuous variables between treatment groups. Chi-Square tests were used, with Cochran correction as appropriate, to compare categorical variables between treatment groups. Statistical Packages for Social Science (SPSS) software version 23.0 for Windows was used for the statistical analysis and a p-value <0.05 was considered statistically significant.

To address confounding factors, a simple logistic regression was done to test the relationship of each variable and the likelihood of experiencing implant-related complications. SPSS software was used to calculate odd ratios and the corresponding confidence intervals. Variables with a p-value <0.20 were chosen for the main effects model in the multivariate analysis.

A note on the terms used in this study: The terms trochanteric and intertrochanteric are used interchangeably to mean all those fractures in AO classification 31A, excluding isolated single trochanter fractures^19^. According to the AO Principles of Fracture Management, subtrochanteric extension is defined as a fracture with its center located below the transverse line at the distal end of the lesser trochanter^20^. Next, the term osteoporosis is used based on the World Health Organization (WHO) diagnostic criteria of bone mineral density (BMD) 2.5 SD or more below the mean for a young-adult reference population or incidence of a fragility fracture in postmenopausal women and men aged 50 years or more^21^.

## RESULTS

A total of 220 patients fulfilled the criteria of this study. Of the 220 patients, 138 (62.7%) underwent short cephalomedullary nailing and 82 (37.3%) underwent long cephalomedullary nailing for the treatment of their intertrochanteric femur fractures.

The characteristics of the patients and their fractures were compared between the two cohorts. Among those variables, the age, pre-injury ADL, and fracture type statistically differed. The median age was significantly higher (p-value = 0.003) in the long CMN group (81 years, IQR 12) compared to the short CMN group (77 years, IQR 14). Significantly more patients who were ADL dependent before the injury were treated with long CMN (p-value = 0.015). For the type of fracture, more AO classification 31A3 fractures were treated with long CMN (p-value <0.001). Other patient characteristics, such as sex, race, previous diagnosis of osteoporosis, ASA classification, mechanism of injury, and presence of other injury, were statistically similar between the two groups (Table I).

**Table I:** Patient characteristics.

**Characteristics**	**Cephalomedullary nail, n (%)**		
	**Short 138**	**Long 82**	**p-value^a^**
Sex			
Female	89 (64.5)	56 (68.3)	0.565
Male	49 (35.5)	26 (31.7)	
Race			
Chinese	68 (49.3)	40 (48.8)	0.728
Indian	37 (26.8)	22 (26.8)	
Malay	30 (21.7)	16 (19.5)	
Others	3 (2.2)	4 (4.9)	
Pre-injury ADL			
Dependent	5 (3.6)	10 (12.2)	0.015
Independent	133 (96.4)	72 (87.8)	
Previous diagnosis of osteoporosis			
Yes	10 (7.2)	7 (8.5)	0.729
No	128 (92.8)	75 (91.5)	
ASA classification			
I	10 (7.2)	1 (1.2)	0.256
II	88 (63.8)	55 (67.1)	
III	39 (28.3)	25 (30.5)	
IV	1 (0.7)	1 (1.2)	
Mechanism of injury			
Fall	130 (94.2)	75 (91.5)	0.294
MVA	5 (3.6)	2 (2.4)	
Unknown	3 (2.2)	5 (6.1)	
Other injury			
Yes	6 (4.3)	6 (7.3)	0.348
No	132 (95.7)	76 (92.7)	
Fracture type (AO classification)			
31A1	80 (60.0)	33 (40.2)	<0.001
31A2	52 (37.7)	30 (36.6)	
31A3	6 (4.3)	19 (23.2)	

Note - ^a^Chi-Square test, p<0.05 as significant at 95% CI

In terms of implants, the Proximal Femoral Nail Antirotation-II [PFNA-II, Synthes Holding AG, Solothurn, Switzerland] was used in the fixation of both groups of patients. The diameter of the nails used (p-value = 0.495), the post-fixation collodiaphyseal angle (p-value = 0.980), and the tip-apex-distance (TAD) index (p-value = 0.221) were statistically comparable between those treated with the long and short CMNs.

Comparing the perioperative outcomes in these two groups, the long CMN cohort showed significantly longer operating time (p-value <0.001), higher intra-operative complications (p-value = 0.010), higher requirement for post-operative blood transfusion (p-value = 0.036), and lower allowance for immediate weight bearing after surgery (p-value <0.001). There was no significant difference in blood loss, change in haemoglobin level, requirement for intra-operative blood transfusion, length of hospital stay after surgery, fracture union, and death (Tables II and III). There was also no significant difference between the two groups in the rate of post-operative complications such as fat embolism syndrome (FES), deep vein thrombosis (DVT), pulmonary embolism (PE), and neurovascular (NV) injury, and implant-related complications including surgical site infection (SSI), screw cut out, implant failure, periprosthetic fracture, or revision surgery (Table IV).

**Table II:** Age and perioperative outcomes, continuous variables.

**Continuous variables**	Test*		**Median (IQR)**	p-value**
	**Short CMN**	**Long CMN**		
Age (years)	Mann-Whitney	77 (14)	81 (12)	0.003
Operating time (minutes)	Mann-Whitney	50 (25)	65 (30)	<0.001
Blood loss volume (ml)	Mann-Whitney	100 (50)	150 (150)	0.058
Change in Hb level (g/dL)	Mann-Whitney	1.20 (1.70)	1.35 (1.40)	0.508
Hospital stays after surgery (days)	Mann-Whitney	3 (2)	3 (3)	0.264
Post-fixation collodiaphyseal angle (degrees)	Independent T	132 (10)	134 (8)	0.980
TAD index	Mann-Whitney	24.85 (8.91)	22.88 (9.60)	0.221

Notes - *Using tests of normality Kolmogorov-Smirnov and Shapiro-Wilk to identify distribution pattern, Mann-Whitney test is used for variables with skewed distribution, and Independent T test is used for variables with normal distribution, **p<0.05 is significant

**Table III:** Perioperative outcomes, categorical variables.

**Categorical variables**	**CMN, n (%)**		
	**Short 138 (62.7)**	**Long 82 (37.3)**	**p-value^a^**
Intra-operative blood transfusion			
Yes	5 (3.6)	8 (9.8)	0.062
No	133 (96.4)	74 (90.2)	
Post-operative blood transfusion			
Yes	28 (20.3)	27 (32.9)	0.036
No	110 (79.7)	55 (67.1)	
Intra-operative complication			
Yes	2 (1.4)	7 (8.5)	0.010
No	136 (98.6)	75 (91.5)	
Allowance for weight bearing after surgery			
Yes	91 (65.9)	22 (26.8)	<0.001
No	47 (34.1)	60 (73.2)	
Fracture union at last follow-up			
Yes	106 (76.8)	61 (74.4)	0.685
No	32 (23.2)	21 (25.6)	
Death			
Yes	17 (12.3)	15 (18.3)	0.224
No	121 (87.7)	67 (81.7)	

Note - ^a^Chi-Square test, p<0.05 as significant at 95% CI

**Table IV:** Complications.

**Characteristics**	**CMN, n (%)**		
	**Short 138 (62.7)**	**Long 82 (37.3)**	**p-value^a^**
Experience either FES, DVT, PE, NV injury, or other complications (post-operative complications)			
Yes	26 (18.8)	19 (23.2)	0.441
No	112 (81.2)	63 (76.8)	
Experience either SSI, screw cut out, implant failure, periprosthetic fracture, or revision surgery (implant-related complications)			
Yes	10 (7.2)	7 (8.5)	0.729
No	128 (92.8)	75 (91.5)	
SSI			
Yes	4 (2.9)	3 (3.7)	0.756
No	134 (97.1)	79 (96.3)	
Screw cut out			
Yes	3 (2.2)	2 (2.4)	0.898
No	135 (97.8)	80 (97.6)	
Implant failure			
Yes	1 (0.7)	1 (1.2)	0.708
No	137 (99.3)	81 (98.8)	
Periprosthetic fracture			
Yes	3 (2.2)	1 (1.2)	0.608
No	135 (97.8)	81 (98.8)	
Revision surgery			
Yes	3 (2.2)	1 (1.2)	0.608
No	135 (97.8)	81 (98.8)	

Note - ^a^Chi-Square test, p<0.05 as significant at 95% CI

This study showed 9 out of 220 cases (4.09%) had intraoperative complications. Among these cases, 2 cases (1.45%) were from the short group, and 7 cases (8.54%) were from the long group; this was statistically significant (p-value = 0.010). One of the cases from the short nail cohort had persistent tachycardia intra-operatively, which required admission to the intensive care unit post-operatively, while the other had prolonged surgery, more than two hours, which required conversion to general anaesthesia. From the long nail cohort, one case had excessive bleeding requiring intra-operative transfusion, two had persistent hypotension intra-operatively, which required admission to the intensive care unit post-operatively, and four had prolonged surgery, more than two hours, which required conversion to general anaesthesia.

Univariate analysis was done to assess the individual effects of each variable on implant-related complications. The simple logistic regression showed that none of the variables were independent predictors for implant-related complications, including those that significantly differ between the short and long CMN groups: age (p-value = 0.257), pre-injury ADL (p-value = 0.874), and fracture type AO classification 31A3 (p-value = 0.114). For multivariate analysis, variables with p-value <0.20 were chosen and forward and backward logistic regression were done, but none of the variables were significant in the main effect model (Table V).

**Table V:** Logistic regression of selected variables for implant-related complications.

**Characteristics**	**Crude odds ratio**	**95% Confidence Interval**		**p-value**
		**Lower Limit**	**Upper Limit**	
CMN type				
Short	1.00	-	-	
Long	1.20	0.44	3.27	0.729
Gender				
Male	1.00	-	-	
Female	0.72	0.26	1.97	0.523
**Age**	**0.98**	**0.94**	**1.02**	**0.257**
Race				
Chinese	1.00	-	-	
Indian	0.15	0.02	1.21	0.075
Malay	0.62	0.16	2.32	0.473
Others	3.53	0.61	20.39	0.159
Pre-injury ADL				
Dependent	0.84	0.10	6.84	0.874
Independent	1.00	-	-	
Fracture type				
31A1	1.00	-	-	
31A2	1.20	0.39	3.70	0.757
31A3	2.88	0.78	10.74	0.114
Operating time	1.01	0.99	1.03	0.249
Blood loss volume	1.00	0.99	1.01	0.130
Intra-operative complications				
Yes	1.52	0.18	12.95	0.700
No	1.00	-	-	
Nail diameter				
9mm	7.56	0.73	78.09	0.090
10mm	1.28	0.15	10.70	0.823
11mm	0.90	0.09	9.17	0.925
12mm	1.00	-	-	

## DISCUSSION

Compared to the long CMN, the short CMN has been shown previously to give a significantly higher risk of secondary fracture due to the formation of stress risers at the tip of the nail^6-8^. Some authors attribute this to the older implant design, and this is supported by the more recent studies showing comparable rates of periprosthetic fractures and other implant-related complications between long and short CMNs^10,14-16^.

This study compares the perioperative outcomes and complications of long and short CMN used in the treatment of osteoporotic intertrochanteric femur fractures. Conducted in a hospital in Malaysia, the study population is Asian, which has a different morphology of the femur compared to their Caucasian counterparts, notably the femoral length and curvature and the thickness of the cortex^22,23^. These differences may influence formation of stress risers and secondary fractures. However, using the PFNA-II which is specifically designed for the Asian population, the findings of this study also show comparable rates of periprosthetic fractures and other implant-related complications (SSI, screw cut out, implant failure, and revision surgery) between the short and long groups. This study also shows none of the variables, whether patient-, fracture-, implant-, or fixation-related are an independent risk factors for implant-related complications. In regards to perioperative outcomes, this study finds significant difference in operating time, postoperative blood transfusion requirement, and immediate allowance for weight bearing after surgery between the long and short CMN.

Similar to other studies, the long CMN group has significantly longer operating time in this study^10,14,15,18,24,25^. This is due the free-hand placement of the distal locking screws and the diaphyseal reaming for the long nail. The long CMN group also has a significantly higher intra-operative complications as listed above, likely due to the longer operating time and the diaphyseal reaming.

Regarding blood loss, although the median estimated blood loss volume is higher for long CMNs compared to short CMNs, it is not statistically significant. The change in haemoglobin levels and intra-operative blood transfusion requirement is also comparable between the two groups. Only the post-operative transfusion requirement is significantly higher for the long CMN cohort. Some studies have shown significantly higher blood loss for long CMN and this is again mainly attributed to the longer operating time and the diaphyseal reaming^15,24,25^.

Patients treated with long CMN were more likely not to be allowed to weight-bear on the operated leg immediately post-surgery compared to those treated with short CMN. This finding is despite both groups had comparable stable reduction and fixation. Therefore, the difference is likely due to patient characteristics as discussed before, in which the long nail cohort had older and more frail patients. It may also be due to the long nail being more frequently used for the less stable fracture configuration, the AO classification 31A3.

Other outcomes such as fixation measures, length of hospital stay, fracture union, post-operative complications, and one-year mortality rate are comparable between the two cohorts.

One of the limitations of this study was its retrospective design. Inherent to this, information that can be obtained was limited to those charted in the records. Moreover, without randomisation, there may be selection bias as the choice of implant was based on the preference of the surgeon. Another limitation is the minimum follow-up of 12 months. Due to this short follow-up, there is a high possibility of missing long-term complications. Lastly, this study had no information on the functional recovery of the patients.

## CONCLUSION

In this study, there was no difference in the fracture union rate, mortality rate, and rates of surgical site infection, screw cut out, implant failure, periprosthetic fracture, and revision surgery between patients treated with long and short CMN. Those treated with short CMN had shorter operating time. Therefore, both the long and short CMNs are safe and reliable options in treating intertrochanteric femur fractures. However, as this study and others have shown, the long nail does not provide extra protection against periprosthetic fractures, and it has the disadvantage of longer operating time.

## CONFLICT OF INTEREST

The authors declare no potential conflict of interest.
